# Global distribution, clinical characteristics, and outcomes of human intestinal capillariasis, 2000–2025: a systematic review

**DOI:** 10.1186/s40249-026-01464-3

**Published:** 2026-06-15

**Authors:** Jurairat Jongthawin, Kinley Wangdi, Frederick Ramirez Masangkay, Manas Kotepui

**Affiliations:** 1 Faculty of Medicine, Mahasarakham University, Maha Sarakham, 44000 Thailand; 2 HEAL Global Research Center, Health Research Institute, Faculty of Health, University of Canberra, Bruce, ACT 2617 Australia; 3 National Centre for Epidemiology and Population Health, College of Law, Governance and Policy, Australian National University, Acton, ACT 2601 Australia; 4 Department of Medical Technology, Faculty of Pharmacy, University of Santo Tomas, 1008 Manila, Philippines; 5 Research Center for the Natural and Applied Sciences, University of Santo Tomas, 1008 Manila, Philippines; 6 Medical Technology Program, Faculty of Science, Nakhon Phanom University, Nakhon Phanom, 48000 Thailand

**Keywords:** *Capillaria philippinensis*, Intestinal capillariasis, Raw fish consumption, Epidemiology, Systematic review

## Abstract

**Background:**

Intestinal capillariasis, caused by *Capillaria philippinensis*, is an uncommon but potentially fatal parasitic infection associated with the consumption of raw or undercooked freshwater fish. Despite its sporadic occurrence, its clinical severity and diagnostic challenges warrant a comprehensive synthesis of available evidence. This systematic review aimed to summarize the geographic distribution, risk factors, clinical manifestations, diagnostic approaches, and treatment outcomes of human intestinal capillariasis.

**Methods:**

A systematic search of six electronic databases (PubMed, Scopus, EMBASE, Web of Science, Nursing & Allied Health Premium, Ovid) was conducted to identify studies published between January 2000 and August 2025. Eligible studies included case reports, case series, and observational studies investigating *C. philippinensis* infection in humans. Study selection was performed independently by two reviewers, while data extraction was conducted by one reviewer using a standardized template and independently verified by a second reviewer, with discrepancies resolved through discussion. Data were extracted and synthesized narratively following the PRISMA 2020 guidelines.

**Results:**

Thirty-two studies involving 348 patients were included, comprising studies from Asia (25/32), Africa (6/32), and South America (1/32). The burden of intestinal capillariasis was high in symptomatic or high-risk groups (5.0–23.0%) but extremely low (< 0.01%) in community-based or routine screening populations. Consumption of raw or undercooked freshwater fish was consistently identified as the principal risk factor. Clinical features were highly consistent across studies, characterized by chronic watery diarrhea, abdominal pain, weight loss, and peripheral edema. Hypoalbuminemia was the hallmark laboratory abnormality, frequently accompanied by electrolyte disturbances and anemia. Diagnosis relied primarily on stool microscopy, although limited sensitivity often required repeated examinations; histopathology and polymerase chain reaction provided important adjunctive confirmation in selected cases. Co-infections with other intestinal parasites were commonly reported. Treatment with albendazole or mebendazole was generally effective, with comparable outcomes between agents; however, delayed diagnosis was associated with severe complications and occasional mortality.

**Conclusions:**

Intestinal capillariasis is an underrecognized foodborne infection with a consistent clinical and laboratory profile across study designs. Early diagnosis through repeated stool examination and timely treatment with benzimidazoles is critical to improving outcomes. Strengthening food safety practices and enhancing surveillance are essential to reduce disease burden.

**Graphical Abstract:**

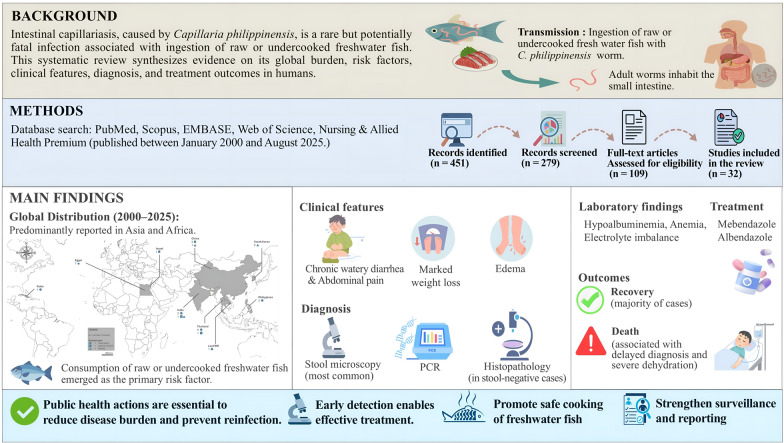

**Supplementary Information:**

The online version contains supplementary material available at 10.1186/s40249-026-01464-3.

## Background

Human intestinal capillariasis is a severe foodborne parasitic disease caused by *Capillaria* (= *Paracapillaria*) *philippinensis*. Although *C. hepatica* is another human-infecting species, it causes hepatic capillariasis and is not involved in intestinal infection [1]. Transmission occurs through ingestion of raw or undercooked freshwater fish containing infective larvae, a dietary practice common in endemic areas (Supplementary Text S1). After ingestion, larvae invade the intestinal mucosa and mature into small adult worms. Female worms produce thick-shelled eggs, excreted in feces, and thin-shelled eggs, which embryonate within the host, enabling autoinfection and, if untreated, potential hyperinfection. Freshwater fish act as intermediate hosts, whereas piscivorous birds have been proposed as possible reservoirs. However, natural infection in birds has not been definitively confirmed, and the role of migratory routes in parasite dissemination remains hypothetical [1].

Clinically, intestinal capillariasis often presents as chronic or intermittent watery diarrhea, abdominal pain, borborygmi, weight loss, and generalized weakness. Severe infections can progress to protein-losing enteropathy, hypoalbuminemia, edema, electrolyte imbalance, septicemia, and death if untreated [2–4]. Early infections may be asymptomatic or masked by co-infections with other intestinal parasites, complicating diagnosis. Delays in diagnosis are common due to nonspecific symptoms, intermittent egg shedding, and limited access to advanced molecular or histopathological testing [3].

The first documented case occurred in the Philippines in 1964, followed by a major outbreak in Northern Luzon between 1965 and 1967, resulting in over 1000 cases and 77 deaths. From 1967–1990, Northern Luzon reported 1884 cases and 110 deaths. Sporadic cases have also been reported in Japan, Thailand, the Republic of Korea, China, Indonesia, Iran, and Egypt [3–5]. Although the overall incidence has declined, recent reports and small-scale studies indicate that intestinal capillariasis remains an underrecognized public health concern [6].

Despite being effectively treatable with benzimidazole derivatives, delayed diagnosis increases the risk of complications and mortality. Traditional consumption of raw fish and occupational exposure remain major risk factors, while globalization, climate change, evolving dietary practices, and human mobility may alter transmission patterns and introduce the parasite to new regions [1, 7]. The present systematic review aims to collate and synthesize the global burden, clinical characteristics, and outcomes of intestinal capillariasis, primarily caused by *C. philippinensis*. The synthesis employs a narrative approach, grouping and integrating quantitative data (burden/demographics) from larger observational studies with clinical characteristics/severity from case reports and case series.

## Methods

### Registration and protocol

This systematic review was conducted in accordance with the Preferred Reporting Items for Systematic Reviews and Meta-Analyses (PRISMA 2020) guidelines. The systematic review protocol was registered in PROSPERO (CRD420251130104).

### Review question

The systematic review focused on studies published between January 2000 and August 2025 that investigated human intestinal capillariasis, primarily caused by *C. philippinensis*. The review was guided by the Population, Exposure, Comparator, and Outcomes (PECO) framework [8]. P—participants residing in endemic areas; E—exposure to *C. philippinensis* infection; C—no predefined comparator at the review level (although some included primary studies employed internal control groups within their own study designs); and O—burden, clinical characteristics, and outcomes.

### Definitions

For the purpose of this review, intestinal capillariasis was classified according to the level of diagnostic confirmation. Confirmed cases were defined as those with at least one of the following: i) Detection of *C. philippinensis* eggs, larvae, or adult worms on stool microscopy; ii) Histopathological identification of parasite stages in intestinal biopsy specimens; iii) Molecular confirmation by polymerase chain reaction (PCR).

Probable cases were defined as patients with compatible clinical manifestations and epidemiologic exposure (e.g., consumption of raw freshwater fish), with supportive but non-definitive findings (e.g., suggestive imaging or endoscopic features) in the absence of direct parasitological or molecular confirmation. Only studies reporting confirmed or clearly documented diagnostic evidence were included in the primary synthesis.

### Eligibility criteria

Studies were considered eligible if they reported human intestinal capillariasis caused by *C. philippinensis*, with infection confirmed through parasitological examination (stool or gastrointestinal content analysis), histopathological identification, or molecular diagnostic methods. Eligible study designs included observational studies (cross-sectional, case-control, retrospective, or prospective), case series, and case reports published between January 2000 and August 2025. Studies were excluded if they involved animal or in vitro experiments, focused on other *Capillaria* species (such as *C. hepatica* or *C. aerophila*), reported environmental detection without confirmed human infection, or were reviews, editorials, letters, book chapters, or conference abstracts lacking original data. Additionally, studies without a confirmed diagnosis of *C. philippinensis* were excluded. No language restriction was applied.

### Search strategy

A comprehensive search was conducted across six electronic databases: PubMed, Scopus, EMBASE, Web of Science, Nursing & Allied Health Premium, and Ovid. The search combined relevant Medical Subject Headings (MeSH) and free-text terms such as “*Capillaria*” and “human”. The general search strategy was as follows: (“*Capillaria philippinensis*” OR Capillarias OR Skrjabinocapillaria OR Skrjabinocapillarias OR “*Paracapillaria philippinensis*” OR “*Aonchotheca philippinensis*”) AND (human OR patients). In addition, Google Scholar was searched as a supplementary source to identify potentially missed studies and grey literature. Due to the large volume of results retrieved and the relevance-based ranking algorithm used by Google Scholar, only the first 200 records sorted by relevance were screened [9]. The specific search strategy was slightly modified for each database, as applicable (Table S1).

### Study selection and data extraction

All retrieved articles were imported into EndNote (version 21.0, Philadelphia, PA, USA) for deduplication. Screening was performed in two stages: (1) title and abstract screening and (2) full-text review. Study selection was independently conducted by two reviewers (MK and JJ), with disagreements resolved through discussion. Data from each included study were extracted using a standardized template covering publication year, country, study design, sample size, participant characteristics, diagnostic methods, clinical manifestations, treatment regimens, and outcomes. Data extraction was conducted by one author (MK) and cross-checked by another (JJ). Any disagreements regarding study selection or data extraction were resolved through discussion until consensus was achieved.

### Risk of bias assessment

Risk of bias for observational studies was assessed using the Joanna Briggs Institute (JBI) critical appraisal tools for cross-sectional, case reports, case series, and case-control studies [10]. Two reviewers (MK, JJ) independently appraised study quality, and discrepancies were resolved by consensus.

### Data synthesis

A narrative synthesis approach was employed to integrate findings from heterogeneous study designs. Quantitative data from observational studies were summarized descriptively, focusing on prevalence (proportion), demographic patterns, and regional distribution. Qualitative synthesis of all studies was performed to characterize clinical features, diagnostic methods, laboratory findings, treatment, and outcomes. Findings were organized thematically to reflect disease burden, risk factors, and clinical presentation and outcomes across included studies.

## Results

### Search results

**Fig. 1:**
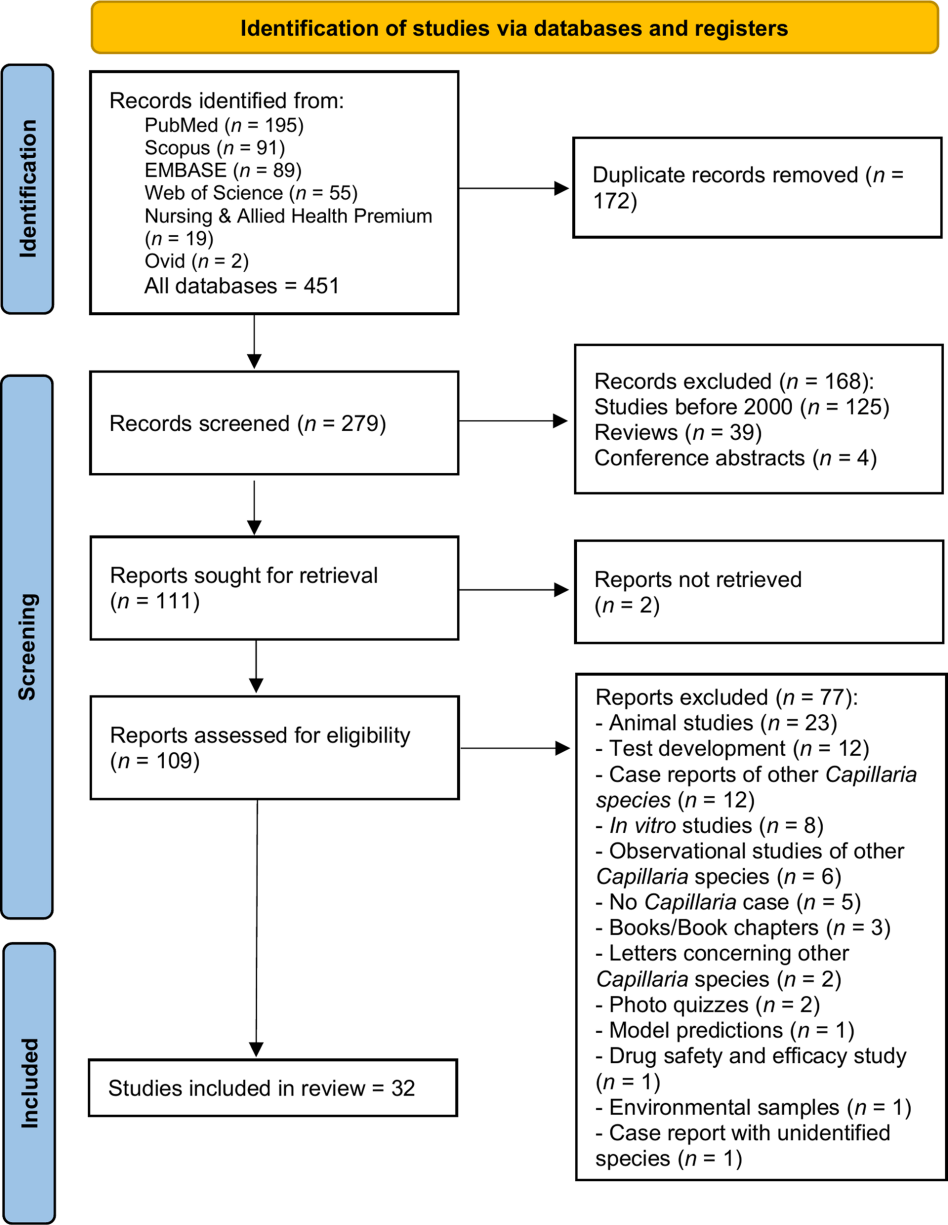
The PRISMA 2020 flow diagram summarizing the selection process of studies included in the systematic review

A total of 451 records were initially identified across six databases; 172 duplicates were removed, leaving 279 records for screening. Following title and abstract screening, 168 records were excluded for being published before 2000, being review articles, or being conference abstracts. Of the 111 reports for retrieval, two could not be obtained, resulting in 109 full-text reports assessed for eligibility. From the eligibility assessment stage, 77 were excluded for reasons including animal or in vitro studies, research on other *Capillaria* species, the absence of *Capillaria* cases, or non-original sources such as books and letters. After screening the first 200 records in Google Scholar, no additional eligible studies were identified beyond those already captured in the primary database searches. In total, 32 studies met the inclusion criteria and were included in the final systematic review (Fig. 1).

### Summary characteristics of included studies

Studies included in this systematic review were published between 2000 and 2024. The literature search covered studies published from January 2000 to August 2025; however, no eligible studies published in 2025 were identified. A total of 32 studies comprising 348 patients with intestinal capillariasis were included. Most were case reports (17/32, 53.1%) [11–27], followed by case series (8/32, 25.0%) [2, 4, 28–33], cross-sectional studies (4/32, 12.5%) [6, 34–36], prospective observational studies (2/32, 6.3%) [37, 38], and one case-control study (1/32, 3.1%) [39]. The majority of studies reported intestinal capillariasis from Asia (25/32 studies) [2, 4, 6, 11, 13–16, 18–20, 22–27, 29, 31–36, 38], followed by Africa (6/32 studies) [12, 17, 28, 30, 37, 39], and South America (1/32 study) [21]. Details of all studies included in the systematic review are listed in Table S2.

### Risk of bias

The majority of included studies were assessed as low risk of bias, particularly case reports, case series, and the case-control study, which generally demonstrated adequate reporting of clinical, diagnostic, and methodological details (Table 1, Table S3).

**Table 1:** Summary of key domains driving risk of bias among included studies

Study (Author, Year)	Study design	Overall risk of bias	Key domains driving risk
Amin et al., 2015 [37]	Prospective observational study	Moderate risk of bias	Unclear exposure measurement Failure to identify and control confounding factors
Praharaj et al., 2017 [38]	Prospective observational study	Low risk of bias	–
Belizario et al., 2000 [34]	Cross-sectional study	Moderate risk of bias	Unclear exposure measurement Failure to identify and control confounding factors
Belizario et al., 2010 [35]	Cross-sectional study	Moderate risk of bias	Unclear exposure measurement Failure to identify and control confounding factors
Prasongdee et al., 2022 [6]	Cross-sectional study	Moderate risk of bias	Unclear exposure measurement Failure to identify and control confounding factors
Wattanawong et al., 2019 [36]	Cross-sectional study	Low risk of bias	–
Bair et al., 2009 [11]	Case report	Low risk of bias	–
El-Karaksy et al., 2004 [12]	Case report	Low risk of bias	–
Fan et al., 2012 [13]	Case report	Low risk of bias	–
Ha et al., 2013 [14]	Case report	Low risk of bias	–
Jung et al., 2012 [15]	Case report	Low risk of bias	–
Kasher et al., 2022 [16]	Case report	Low risk of bias	–
Khalifa et al., 2000 [17]	Case report	Low risk of bias	–
Kusolsuk et al., 2008 [18]	Case report	Low risk of bias	–
Lee et al., 2024 [19]	Case report	Low risk of bias	–
Manalo et al., 2012 [20]	Case report	Low risk of bias	–
Núñez et al., 2010 [21]	Case report	Low risk of bias	–
Rana et al., 2009 [22]	Case report	Low risk of bias	–
Sangchan et al., 2007 [23]	Case report	Low risk of bias	–
Srisajjakul et al., 2022 [24]	Case report	Moderate risk of bias	Patient history timeline unclear Incomplete reporting of adverse events Insufficient description of intervention and post-treatment clinical course
Thewjitcharoen et al., 2012 [25]	Case report	Low risk of bias	–
Vasantha et al., 2012 [26]	Case report	Low risk of bias	–
Wongsawasdi et al., 2002 [27]	Case report	Low risk of bias	–
Attia et al., 2012 [28]	Case series	Low risk of bias	–
Bair et al., 2001 [29]	Case series	Low risk of bias	–
Bair et al., 2004 [4]	Case series	Low risk of bias	–
El-Dib et al., 2020 [30]	Case series	Low risk of bias	–
Limsrivilai et al., 2014 [31]	Case series	Low risk of bias	–
Lu et al., 2006 [32]	Case series	Low risk of bias	–
Sadaow et al., 2018 [2]	Case series	Low risk of bias	–
Soukhathammavong et al., 2008 [33]	Case series	Moderate risk of bias	Incomplete or unclear participant inclusion
Waly et al., 2021 [39]	Case-control study	Low risk of bias	–

– No key domains contributing to risk of bias were identified based on the assessment criteria

Among observational studies (cross-sectional and prospective designs), several were rated as moderate risk of bias [6, 34, 35, 37], primarily due to unclear exposure measurement and failure to identify or control for confounding factors.

Only two descriptive studies were rated as moderate risk [24]: one case report due to incomplete reporting of patient history, intervention details, and adverse events [24], and one case series due to unclear or incomplete participant inclusion, raising concerns about potential selection bias [33].

### Global distribution and evidence of intestinal capillariasis

The geographic distribution of intestinal capillariasis from the included studies is shown in Table 2, summarizing country, provincial-level administrative divisions, and specific localities where available. Characteristics of patients with intestinal capillariasis reported from the included studies are shown in Table 3. Of the six studies, four cross-sectional studies were conducted in Asia [6, 34–36]. Additionally, two prospective observational studies were conducted in Africa [37] and Asia [38].

**Table 2:** Geographic distribution of intestinal capillariasis cases from included studies

Country	Provincial-level administrative division(s)	City/local area(s) reported	References
Egypt	Beni-Suef Governorate	–	[37]
	El-Menia Governorate	–	[12]
	Assiut Governorate	–	[17, 28]
	Cairo, Beni Suef	–	[30]
Philippines	Davao de Oro	Monkayo	[34]
	Mindanao	Barangay Matam, Barangay Dabiak, Barangay Carupay	[35]
	Occidental Mindoro	Santa Cruz	[20]
India	Vellore	–	[38]
	Andhra Pradesh state	–	[26]
	–	–	[22]
Thailand	Khon Kaen	–	[2, 6, 23]
	76 provinces (except Bangkok)	–	[36]
	Prachin Buri	–	[18]
	Nakhon Sawan	–	[25]
	Central Thailand	–	[27]
	Bangkok	–	[31]
	–	–	[24]
China	Taiwan	–	[4, 11, 19, 32]
	Hainan	Danzhou	[13]
	Taiwan	Tai-tung	[29]
The Republic of Korea	–	–	[14]
	Gyeongsangnam-do	Sacheon-si	[15]
Lao PDR	Vientiane	–	[33]
Israel	–	–	[16]
Cuba	Centro Habana	Havana	[21]

– Not specified

**Table 3:** Characteristics of patients with intestinal capillariasis reported from the included studies

Patient characteristic	Parameter	Case-control study (*n*/*N*)	Case reports (*n*/*N*)	Case series (*n*/*N*)	Prospective observational studies (*n*/*N*)	Cross-sectional studies (*n*/*N*)	References
Origin	Asia	–	14/17	6/8	1/2	4/4	[2, 4, 6, 11, 13–16, 18–20, 22–27, 29, 31–36, 38]
	Thailand	–	5/17	2/8	1/2	1/4	[2, 6, 18, 23–25, 27, 31, 38]
	Philippines	–	1/17	–		2/4	[20, 34, 35]
	India	–	2/17	–	1/2	–	[22, 26, 38]
	China	–	3/17	3/8	–	–	[4, 11, 13, 19, 29, 32]
	The Republic of Korea	–	2/17	–	–	–	[14, 15]
	Israel	–	1/17	–	–	–	[16]
	Lao PDR	–	0/17	1/8	–	–	[33]
	Africa: Egypt	1/1	2/17	2/8	1/2	–	[12, 17, 28, 30, 37, 39]
	South America: Cuba	–	1/17	–	–	–	[21]
Eating history	Half-cooked or half-boiled fish; grilled fish; raw parts such as caviar (gravid uterus); small salted fish	–	–	–	1/2	–	[37]
	Undercooked fish and shellfish; sashimi made from raw *Misgurnus anguillicaudatus* (loach)	–	1/17	–	–	–	[13]
	Raw fish, particularly the common blackish goby (*Acanthogobius flavimanus*)	–	1/17	–	–	–	[15]
	Raw freshwater fish locally known as “Phra-Pla Siw/Soi”	–	1/17	–	–	–	[18]
	Traditional dishes such as “dog” and “kilawin”	–	1/17	–	–	–	[20]
	Breaded fish purchased from street vendors	–	1/17	–	–	–	[21]
	Raw or insufficiently cooked freshwater fish or prawns	–	–	4/8	–	1/4	[2, 29, 31–33]
Occupations	Housewives	–	1/17	3/8	1/2	–	[4, 17, 28, 29, 37]
	Students	–	–	–	1/2	–	[37]
	Fishermen	–	2/17	2/8	–	–	[4, 14, 20, 29]
	Workers	–	1/17	–	–	–	[16]
	Migrant workers	–	–	1/8	–	–	[33]
	Dried bamboo shoot processors	–	1/17	–	–	–	[19]
	Tricycle drivers	–	1/17	–	–	–	[20]
	Construction workers	–	1/17	–	–	–	[25]
	Farmers	–	1/17	2/8	–	–	[4, 20, 29]
	Schoolteachers	–	–	1/8	–	–	[28]
	Merchants	–	–	1/8	–	–	[4]
	Individuals whose fathers were fishermen who regularly consumed fish	–	1/17	–	–	–	[12]
Clinical signs and symptoms	Chronic watery diarrhea	–	15/17	6/8	1/2	2/4	[4, 11–15, 18–32, 34, 35, 37]
	Abdominal or colicky pain and cramps	–	10/17	6/8	1/2	1/4	[2, 4, 11, 13–15, 17, 18, 20, 23, 25, 27, 29–32, 35, 37]
	Gurgling or borborygmi	–	2/17	6/8	1/2	1/4	[2, 11, 28–34, 37]
	Marked weight loss or emaciation	–	11/17	6/8	1/2	1/4	[2, 4, 11, 13–15, 17–20, 22, 25, 28–32, 34, 37]
	Peripheral edema (lower limbs, face, or scrotum)	–	11/17	6/8	1/2	1/4	[2, 11–13, 15, 16, 20, 22, 23, 25–28, 30–34, 37]
	Comatose state and bilateral pleural effusion	–	–	1/8	–	–	[28]
Laboratory alterations	Hypoalbuminemia	–	15/17	8/8	1/2	–	[2, 4, 11–16, 18–20, 22–33, 37]
	Hyponatremia and hypokalemia	–	5/17	2/8	1/2	–	[4, 11, 14, 20, 23, 26, 29, 37]
	Hypokalemia without hyponatremia	–	3/17	–	–	–	[22, 25, 27]
	Peripheral eosinophilia	–	4/17	–	–	–	[21, 24–26]
	Normal leukocyte counts or leukocytosis without eosinophilia	–	3/17	–	–	–	[14, 23, 27]
	Leukocytosis	–	2/17	1/8	–	–	[14, 19, 30]
	Leukopenia	–	1/17	–	–	–	[25]
	Low hemoglobin levels, mild anemia, or microcytic anemia	–	8/17	6/8	–	–	[2, 4, 12, 13, 16, 19, 22, 23, 25, 27, 28, 30–32]
	Folate deficiency; iron deficiency anemia	–	–	1/8	–	–	[31]
	Malabsorption of fats or carbohydrates	–	–	2/8	–	–	[2, 4]
Detection methods	Direct smear and/or concentration methods	1/1	10/17	7/8	2/2	4/4	[2, 4, 6, 12, 13, 17–19, 21, 24–32, 34–39]
	Rapid immunochromatographic test (ICT)	–	–	–	–	1/4	[6]
	Scanning electron microscopy (SEM)	–	–	1/8	–	–	[28]
	Polymerase chain reaction (PCR)	–	2/17	–	–	–	[15, 16]
	Chest X-ray	–	4/17	2/8	–	–	[12, 14, 23, 26, 30, 31]
	Ultrasonography	–	3/17	3/8	1/2	–	[12, 21, 26, 28, 30, 33, 37]
	Endoscopy	–	4/17	–	–	–	[15, 17, 22, 27]
	Esophagogastroduodenoscopy (EGD)	–	2/17	–	–	–	[11, 15]
	Jejunoscopy	–	1/17	–	–	–	[21]
	Gastroscopy	–	2/17	1/8	–	–	[16, 25, 33]
	Colonoscopy	–	8/17	2/8	–	–	[11, 14–16, 19, 22, 25, 27, 29, 31]
	Enteroscopy	–	3/17	2/8	–	–	[22–24, 30, 31]
	Computed tomography (CT)	–	4/17	1/8	–	–	[14–16, 25, 31]
Developmental stages of *C. philippinensis*	Only eggs	–	7/17	–	1/2	1/4	[12, 13, 19–21, 23, 27, 34, 38]
	Only larvae	–	2/17	–	–	–	[15, 16]
	Both eggs and larvae	–	1/17	–	–	–	[12]
	Eggs and adult worms	–	2/17	–	–	–	[18, 25]
	Multiple developmental stages- eggs, larvae, and/or adult worms	–	2/17	–	–	1/4	[6, 17, 22]
	The presence of eggs at the time of diagnosis, with adult worms subsequently detected during treatment	–	1/17	–	–	–	[26]
	Adult worms were detected only on the second or third day following a provocative test	–	–	–	1/2	–	[37]
Histopathological findings	Flattened jejunal villi with crypt hyperplasia	–	1/17	–	–	–	[27]
	Mild villous atrophy with nonspecific inflammatory infiltration	–	1/17	–	–	–	[12]
	Chronic inflammation with mucosal atrophy and eosinophilic infiltration	–	2/17	–	–	–	[14, 22]
	Villous atrophy with intense plasma cell infiltration	–	1/17	–	–	–	[15]
	Active inflammation with severe villous atrophy and infiltration of plasma cells and eosinophils	–	1/17	–	–	–	[16]
	Chronic inflammation without villous atrophy	–	1/17	–	–	–	[17]
	Severe partial villous atrophy with increased intraepithelial lymphocytes (findings consistent with intestinal malabsorption syndrome)	–	1/17	–	–	–	[21]
Co-infections	*Giardia lamblia*	1/1	1/17	1/8	1/2	–	[12, 33, 37, 39]
	*Entamoeba histolytica*	1/1	1/17	1/8	1/2	–	[20, 33, 37, 39]
	*Cryptosporidium parvum*	1/1	–	–	1/2	–	[37, 39]
	*Trichomonas* spp.	–	–	1/8	–	–	[33]
	*Blastocystis hominis*	1/1	–	–	–	–	[39]
	Microsporidia	1/1	–	–	–	–	[39]
	*Strongyloides stercoralis*	–	1/17	4/8	–	–	[4, 20, 29, 31, 33]
	*Clonorchis sinensis*	–	–	2/8	–	–	[4, 29]
	Hookworm	–	1/17	2/8	–	–	[19, 31, 33]
	*Opisthorchis viverrini*	–	–	2/8	–	–	[31, 33]
	*Hymenolepis nana*	1/1	–	–	–	–	[39]
	*Trichuris trichiura*	–	1/17	–	–	–	[33]
	*Candida albicans*	–	1/17	–	–	–	[20]
	*Mycobacterium tuberculosis* (tuberculous colitis)	–	1/17	–	–	–	[20]
	*Acinetobacter baumannii*	–	1/17	–	–	–	[25]
Treatment	Mebendazole	–	7/17	2/8	–	–	[4, 11, 18, 19, 21, 22, 25, 27, 29]
	Albendazole	–	7/17	3/8	–	–	[4, 12–16, 23, 26, 28, 33]
	Metronidazole	–	2/17	–	–	–	[17, 20]
	Supportive care, including fluid hydration, electrolyte replacement, and antidiarrheal therapy	–	1/17	–	–	–	[11]
	Fluid and electrolyte replacement with protein and multivitamin supplementation	–	1/17	–	–	–	[26]
Outcomes	Prompt recovery	–	15/17	5/8	–	–	[2, 4, 11–23, 26, 27, 29, 32, 33]
	Weight gain	–	4/17	–	–	–	[12, 14, 23, 27]
	Resolution of diarrhea	–	5/17	–	–	–	[12, 19, 20, 23, 26]
	Reduction of edema	–	1/17	–	–	–	[12]
	Normalization of laboratory parameters (total serum protein and albumin levels)	–	2/17	–	–	–	[15, 16]
	Reinfection after resuming consumption of raw fish	–	–	1/8	–	–	[31]
	Death due to septic shock	–	1/17	–	–	–	[12]
	Rapid clinical deterioration leading to death after 10 days in the intensive care unit	–	1/17	–	–	–	[25]
	Prompt recovery in 20 cases, with one fatality	–	–	1/8	–	–	[28]

*–* No evidence, *n/N* number of studies (or reports) in which the characteristic was reported/total number of studies of that study design included in the review

Capillariasis prevalence in the symptomatic population or high-risk participants, i.e., patients presenting with chronic or persistent diarrhea ranged from 5.0–23.0% [6, 34, 35, 37]. The highest prevalence was reported in Thailand (22.6%, 66/292 cases) [6], followed by the Philippines (22.2%, 16/72 cases) [34]. Other studies in symptomatic participants reported 4.9% (10/205 cases) in the Philippines [35], and 5.0% (8/160 cases) in Egypt [37].

In studies in which stool samples were submitted for routine diagnostic examination without restriction to a defined high-risk clinical status, a markedly lower prevalence was observed. One study from India reported less than 0.01% (1/257,588 cases) [38]. Similarly, a community-based study from Thailand reported a prevalence of less than 0.01% (1/16,187 cases) among healthy individuals [36]. Beyond prevalence studies, case series were reported from Egypt [28, 30], China [4, 29, 32], Lao PDR [33], and Thailand [2, 31]. In addition, case-control investigations were conducted in Egypt [39]. Case reports documented intestinal capillariasis in several countries across Asia, including China [11, 13, 19], the Republic of Korea [14, 15], Israel [16], Thailand [18, 23–25, 27], the Philippines [20], and India [22, 26]; in Africa, particularly Egypt [12, 17]; and in South America, such as Cuba [21].

### Risk of bias and interpretation of reported prevalence estimates

Studies conducted in community-based settings [36] or those using stool samples submitted to parasitology laboratories for routine diagnostic screening [38] were assessed as having a lower risk of bias regarding population representativeness. These studies, characterized by broader sampling frames, reported markedly lower prevalence estimates (< 0.01%).

In contrast, studies conducted among symptomatic or referral-based clinical populations [6, 34, 35, 37] were judged to have a moderate risk of bias due to non-representative sampling. The highest reported prevalence estimates (> 20%) were derived from selected high-risk symptomatic participants and were assessed as having moderate risk of bias, primarily due to selection bias.

Case series and case reports were not considered appropriate for estimating prevalence because they lack defined population denominators and are susceptible to referral and selection biases.

### Age, gender distribution, and raw fish consumption

Intestinal capillariasis was reported across a wide age range, from infancy to advanced age (less than 1 year to 98 years), as reported in the majority of included studies (reported in 25/32 studies) [2, 4, 6, 11–29, 31–39]. Gender distribution was inconsistent across observational study designs. Among prospective observational studies, one study reported a strong female predominance (100.0%, 8/8 cases) [37], whereas another documented a single male case (1/2 studies) [38]. Similarly, cross-sectional studies demonstrated variable distributions, with most reporting a relatively balanced proportion of males (50.0–60.0% male cases in 3/4 studies) [6, 34, 35], while one study reported a single male case (1/4 studies) [36].

Male predominance was observed in the majority of case series. Sex distribution was reported in 7/8 case series [2, 4, 28, 29, 31–33], of which most demonstrated higher proportions of males (6/8 studies), ranging from 60.0–77.6% [2, 4, 29, 31–33]. In contrast, one study reported a markedly lower proportion of male patients [28]. Male predominance was observed in the majority of case reports (11/17 studies) [11, 14, 15, 18–21, 23–25, 27], whereas female patients accounted for 23.5% (4/17 studies) [12, 13, 17, 26]. Similarly, the case-control study demonstrated a higher proportion of males in both diabetic patients (63.9%) and the control group (78.6%) [39].

Consuming raw or undercooked freshwater fish was the principal exposure risk across study designs. This exposure was reported in 5/17 case reports [13, 15, 18, 20, 21] and 5/8 case series [2, 29, 31–33], as well as in 1/2 prospective observational studies [37]. Reported exposures included consumption of loach sashimi (*Misgurnus anguillicaudatus*) [13], raw fish, particularly the common blackish goby (*Acanthogobius flavimanus*) [15], raw freshwater fish locally known as “Phra-Pla Siw/Soi” [18], traditional dishes such as “dog” and “kilawin” [20], and breaded fish purchased from street vendors [21]. However, the absence of raw fish consumption was also reported, with one case series (1/8 studies) [28] and one case report (1/17 cases) reporting no history of such exposure [11].

### Occupations

One prospective observational study (1/2 studies) reported that patients were housewives and students [37]. Among case series, housewives were reported in three studies (3/8 case series) [4, 28, 29], and fishermen in two studies (2/8 case series) [4, 29]. Other occupations included a migrant worker (1/8 case series) [33], farmers (2/8 case series) [4, 29], a schoolteacher (1/8 case series) [28], and a merchant (1/8 case series) [4].

Among case reports, occupations included housewives (1/17 case reports) [17], fishermen (2/17 case reports) [14, 20], a dried bamboo shoot processor (1/17 case reports) [19], a tricycle driver (1/17 case reports) [20], a construction worker (1/17 case reports) [25], and a farmer (1/17 case reports) [20]. One case report also noted that the patient’s father was a fisherman and that the patient consumed fish at least twice weekly (1/17 case reports) [12].

### Clinical signs and symptoms

Chronic watery diarrhea was the most consistent clinical manifestation across all study designs, reported in 15/17 case reports [11–15, 18–27], 6/8 case series [4, 28–32], 2/4 cross-sectional studies [34, 35], one case-control study (1/1 study) [39], and 1/2 prospective observational studies [37].

Abdominal pain or colicky cramps were also frequently observed, occurring in 10/17 case reports [11, 13–15, 17, 18, 20, 23, 25, 27], 6/8 case series [2, 4, 29–32], 2/4 cross-sectional studies [34, 35], the case-control study (1/1 study) [39], and 1/2 prospective observational studies [37].

Marked weight loss or emaciation and borborygmi were commonly reported symptoms, with weight loss documented in 10/17 case reports [11, 13–15, 17–20, 22, 25], 7/8 case series [2, 4, 28–32], 1/4 cross-sectional studies [34], and 1/2 prospective observational studies [37], while borborygmi was reported in 1/17 case reports [11], 7/8 case series [2, 28–33], 1/4 cross-sectional studies [34], and 1/2 prospective observational studies [37].

Peripheral edema was another frequent clinical feature, observed in 11/17 case reports [11–13, 15, 16, 20, 22, 23, 25–27], 6/8 case series [2, 28, 30–33], 1/4 cross-sectional studies [34], and 1/2 prospective observational studies [37]. Severe manifestations such as coma and bilateral pleural effusion were rarely reported (1/8 case series) [28].

### Laboratory results

Hypoalbuminemia was the hallmark laboratory abnormality across all study designs, reported in 15/17 case reports [11–16, 18–20, 22–27], all case series (8/8 studies) [2, 4, 28–33], and 1/2 prospective observational studies [37]. Electrolyte disturbances, particularly hypokalemia and hyponatremia, were also frequently observed. Combined hyponatremia and hypokalemia were reported in 5/17 case reports [11, 14, 20, 23, 26], 2/8 case series [4, 29], and 1/2 prospective observational studies [37], while hypokalemia without hyponatremia was reported in 3/17 case reports [22, 25, 27] and 3/8 case series [2, 28, 31].

Anemia was another common finding, with low hemoglobin levels or mild to microcytic anemia reported in 8/17 case reports [12, 13, 16, 19, 22, 23, 25, 27] and 6/8 case series [2, 4, 28, 30–32], while nutritional deficiencies such as folate or iron deficiency were less frequently described (1/8 case series) [31]. Hematologic abnormalities were variable, including leukocytosis or leukopenia reported in 3/17 case reports [14, 19, 25] and 1/8 case series [30], as well as normal leukocyte counts in 3/17 case reports [15, 23, 26]. Peripheral eosinophilia was inconsistently observed, reported in 4/17 case reports [21, 24–26]. Evidence of malabsorption was reported in a minority of studies, including impaired absorption of fats or carbohydrates in 2/8 case series [2, 4].

### Detection of *Capillaria philippinensis* in stool specimen and pathological examination

Stool microscopy was the primary diagnostic method across all study designs. It was used in 10/17 case reports [12, 13, 17–19, 21, 24–27], 7/8 case series [2, 4, 28–32], all cross-sectional studies (4/4 studies) [6, 34–36], and both prospective observational studies (2/2 studies) [37, 38]. Repeated stool examinations were required in a minority of cases (2/17 case reports) to detect eggs, larvae, or adult worms [25, 27].

Histopathological examination served as an important alternative diagnostic modality, particularly in cases with negative stool findings. It was reported in 1/17 case reports [16] and 3/8 case series [4, 31, 32]. In some instances, diagnosis was established exclusively through histopathology despite negative stool microscopy and molecular testing [16]. Molecular confirmation using PCR was infrequently reported but demonstrated diagnostic value. PCR was used in 2/17 case reports [15, 16], including cases where stool microscopy was negative (1/17 case reports) [15].

Imaging and endoscopic modalities were commonly used as supportive diagnostic tools rather than for definitive diagnosis. These included ultrasonography (3/8 case series) [28, 30, 33], computed tomography (4 case reports and 1 case series) [14–16, 25, 31], chest X-ray (4 case reports and 2 case series) [12, 14, 23, 26, 30, 31], and endoscopic procedures such as enteroscopy (3 case reports and 1 case series) [22–24, 31] and conventional endoscopy (4 case reports and 2 case series) [15, 17, 22, 27, 30, 31]. Confirmatory and supportive diagnostic modalities for intestinal capillariasis, along with their potential limitations, are shown in Table 4.

**Table 4:** Confirmatory and supportive diagnostic modalities for intestinal capillariasis and their potential limitations

Modality	Typical finding	Diagnostic role	Limitation
Stool microscopy	Eggs/larvae/adults	Confirmatory	May be false-negative
Histopathology	Parasite stages in the mucosa	Confirmatory	Invasive
PCR	Parasite DNA	Confirmatory	Limited availability
Imaging/CT/US	Bowel wall thickening	Supportive	Non-specific
Endoscopy	Villous atrophy	Supportive/biopsy guidance	Cannot confirm species

*PCR* polymerase chain reaction, *DNA* deoxyribonucleic acid, *CT* computed tomography, *US* ultrasonography

### Developmental stages of *Capillaria philippinensis*

Detection of parasite eggs was the most common parasitological finding across study designs. Eggs were identified in 7/17 case reports [12, 13, 19–21, 23, 27], 1/4 cross-sectional studies [34], and 1/2 prospective observational studies [38]. Multiple developmental stages (eggs, larvae, and/or adult worms) were also reported, although less consistently. These were identified in 2/17 case reports [17, 22], 1/4 cross-sectional studies [6], and 1/2 prospective observational studies [37]. Larval stages alone were infrequently detected, reported in 2/17 case reports [15, 16]. Mixed-stage infections were variably reported in case reports, including detection of both eggs and larvae (1/17 case reports) [12] and eggs with adult worms (2/17 case reports) [18, 25]. In one case, eggs were identified at diagnosis, followed by detection of adult worms during treatment (1/17 case reports) [26].

### Histopathological findings

Histopathological abnormalities were primarily reported in case reports and inconsistently assessed across study designs. Histopathological findings were described in 8/17 case reports [12, 14–17, 21, 22, 27], while available case series reported limited or negative findings (2/8 case series) [31, 33].

Villous atrophy with inflammatory infiltration was the most common histopathological feature, identified in 6/17 case reports, including findings of partial to severe villous atrophy, mucosal atrophy, and infiltration by plasma cells and eosinophils [12, 14–16, 22, 27].

Additional inflammatory changes were also reported, including chronic inflammation with or without villous atrophy (2/17 case reports) [17, 21] and crypt hyperplasia (1/17 case reports) [27]. Detection of parasite structures on histopathology was inconsistent, with no identifiable developmental stages of *C. philippinensis* observed in 1/17 case reports [25] and in 2/8 case series [31, 33].

### Co-infections

Co-infections with other intestinal pathogens were reported across all study designs. These were identified in 4/17 case reports [12, 19, 20, 25], 4/8 case series [4, 29, 31, 33], 1/2 prospective observational studies [37], and the case-control study (1/1 study) [39].

Helminth co-infections were among the most frequently reported, particularly *Strongyloides stercoralis*, which was identified in 1/17 case reports [20] and 4/8 case series [4, 29, 31, 33]. Other helminths included *Clonorchis sinensis* (2/8 case series) [4, 29], hookworm (1/17 case reports and 2/8 case series) [19, 31, 33], *Opisthorchis viverrini* (2/8 case series) [31, 33], and *Trichuris trichiura* (1/8 case series) [33].

Protozoan co-infections were also observed across study designs, including *Giardia lamblia* (1/17 case reports, 1/8 case series, 1/2 prospective observational studies, and the case-control study) [12, 33, 37, 39], *Entamoeba histolytica* (1/17 case reports, 1/8 case series, and 1/2 prospective observational studies) [20, 33, 37], and *Cryptosporidium parvum* (1/2 prospective observational studies and the case-control study) [37, 39]. Other protozoa, such as *Blastocystis hominis*and *Trichomonas* spp., as well as obligate intracellular parasitic fungi, such as Microsporidia [33, 39].

Additionally, co-infections with a primary pathogen, such as *Mycobacterium tuberculosis*, and opportunistic pathogens, such as *Candida albicans* and *Acinetobacter baumannii*, were each reported in 1/17 case reports [20, 25].

### Treatment

Albendazole and mebendazole were the most commonly used treatments across study designs. Albendazole was administered in 7/17 case reports [12–16, 23, 26] and 3/8 case series [4, 28, 33], while mebendazole was used in 7/17 case reports [11, 18, 19, 21, 22, 25, 27] and 2/8 case series [4, 29]. Metronidazole was used less frequently, reported in 2/17 case reports [17, 20].

Treatment regimens were generally consistent across studies, with mebendazole typically administered at 200 mg twice daily or 400 mg per day for 10–30 days (5/17 case reports and 1/8 case series) [11, 18, 21, 22, 25] [29], and albendazole at 200 mg twice daily or 400 mg per day for 3–30 days (7/17 case reports and 1/8 case series) [12–16, 23, 26, 33]. Variations in dosing and duration were also reported, including lower-dose regimens (1/17 case reports) [19], and pediatric adjustments (1/8 case series) [28].

Supportive management was reported in some case reports (3/17 studies), including fluid resuscitation, electrolyte replacement, and nutritional support [11, 12, 26].

### Outcomes

Clinical recovery following antiparasitic treatment was the predominant outcome across study designs. Favorable outcomes were reported in 15/17 case reports [11–23, 26, 27] and 5/8 case series [2, 4, 29, 32, 33]. Both albendazole and mebendazole demonstrated comparable effectiveness. Albendazole demonstrated favorable outcomes, with successful outcomes reported in 8/17 case reports [12–16, 22, 23, 26] and 4/8 case series [2, 4, 29, 33]. Mebendazole demonstrated favorable outcomes, with recovery reported in 6/17 case reports [11, 17–19, 21, 27] and 4/8 case series [2, 4, 29, 32]. One additional case series reported recovery in most patients (20/21 cases) despite one fatality [28].

Symptomatic and clinical improvements were consistently observed among recovered patients, including resolution of diarrhea (5/17 case reports) [12, 19, 20, 23, 26], weight gain (4/17 case reports) [12, 14, 23, 27], reduction of peripheral edema (1/17 case reports) [12], and normalization of laboratory parameters such as serum protein and albumin levels (2/17 case reports) [15, 16].

Adverse outcomes were rarely reported, with mortality reported in 2/17 case reports [12, 25] and in one case series (1/8 studies) [28].

## Discussion

Despite decades of recognition as a relatively rare parasitic disease, intestinal capillariasis remains a challenge in clinical practice and continues to pose a public health concern, particularly in parts of Asia and Africa. To the best of our knowledge, this systematic review provides a comprehensive synthesis of the geographic distribution, risk factors, clinical manifestations, diagnostic approaches, and treatment outcomes of intestinal capillariasis across multiple study designs.

Intestinal capillariasis has been reported primarily from Asia, including Thailand [2, 6, 18, 23–25, 27, 31, 36], the Philippines [20, 34, 35], China [4, 11, 19, 29, 32], India [22, 26, 38], and the Lao PDR [33], and a few from African countries, especially Egypt [37]. The count-based analysis of prevalence studies demonstrated that observed prevalence varied widely across study populations, rather than solely by geographic location. Studies conducted among symptomatic or clinically selected populations reported higher proportions of infection, reaching approximately 5.0–23.0% [6, 34, 35, 37], whereas studies involving community-based or routine diagnostic samples reported markedly lower proportions (< 0.01%) [36, 38], indicating that observed burden is strongly influenced by sampling strategy rather than geography alone.

Sporadic cases reported outside traditionally recognized endemic areas, including a case reported from Cuba [21], suggest the possibility of broader geographic distribution. However, these observations are based primarily on isolated case reports and therefore should be interpreted cautiously. Migratory birds have been proposed as a potential mechanism for parasite dispersal because they may acquire infection by consuming infected fish and subsequently excrete eggs along migratory routes [40]. However, the evidence supporting this ecological pathway remains limited, and further studies are required to clarify its epidemiological significance.

Intestinal capillariasis affected a wide age range, from infancy to 98 years, reported in 25/32 included studies, indicating that exposure occurs broadly across populations. Previous studies suggest that transmission may occur within households where fish preparation practices are shared [37, 40], potentially explaining infections reported among children and family members of affected individuals. Cultural dietary practices involving raw fish consumption may further contribute to exposure across generations, including younger age groups [35].

Sex distribution showed a predominantly male pattern across descriptive study designs. Male predominance was observed in 6/8 case series and 11/17 case reports, as well as in the case–control study, which reported higher proportions of males in both the patient (63.9%) and control groups (78.6%). In contrast, evidence from observational studies was inconsistent, with some reporting female predominance, particularly in African settings. These variations suggest that sex distribution is likely influenced by behavioral and sociocultural factors rather than biological susceptibility. Differences in dietary practices, such as a higher likelihood of raw fish consumption among men in certain settings [37], and gender roles in food preparation [12], particularly among housewives in endemic regions, may contribute to the observed patterns. Environmental and socioeconomic factors, including rural residence [37] and reliance on locally sourced freshwater fish [34], may further influence exposure risk.

Occupational data were inconsistently reported and derived mainly from descriptive studies. Housewives (3/8 case series; 1/17 case reports) and fishermen (2/8 case series; 2/17 case reports) were the most commonly reported groups, with other occupations less frequently described. One prospective study (1/2 studies) also included housewives and students. These findings suggest that individuals involved in fish handling, preparation, or frequent consumption may be at increased risk; however, the evidence remains inconsistent and largely observational.

Consumption of raw or undercooked freshwater fish was consistently identified as the principal transmission route across study designs, reported in 5/17 case reports, 5/8 case series, and 1/2 prospective observational studies. These findings strongly support foodborne transmission as the dominant pathway. Cultural dietary practices, including consumption of raw fish dishes such as kilawin and Phra-Pla Siw/Soi, remain central risk factors. Although some patients denied such exposure, this was uncommon (1/17 case reports and 1/8 case series), suggesting possible recall bias or unrecognized exposure.

Clinical manifestations were highly consistent across all study designs. Chronic watery diarrhea was the hallmark symptom, reported in 15/17 case reports, 6/8 case series, 2/4 cross-sectional studies, 1/2 prospective studies, and the case-control study. Other frequently reported symptoms included abdominal pain (10/17 case reports and 6/8 case series), weight loss (10/17 case reports and 7/8 case series), borborygmi (7/8 case series), and peripheral edema (11/17 case reports and 6/8 case series). The consistency of these findings across multiple study designs reinforces a recognizable clinical syndrome characterized by chronic diarrhea and protein-losing enteropathy. Severe complications, including systemic manifestations such as pleural effusion or coma, were reported only rarely and primarily in descriptive case series [28].

Laboratory findings were similarly consistent. Hypoalbuminemia emerged as the hallmark laboratory abnormality, reported in 15/17 case reports, all case series (8/8), and 1/2 prospective studies, reflecting severe protein loss. Electrolyte disturbances, particularly hypokalemia and hyponatremia, were also frequently observed across study designs. Anemia was reported in 8/17 case reports and 6/8 case series, likely due to malabsorption and nutritional deficiencies [2, 4]. In contrast, eosinophilia was uncommon (4/17 case reports), possibly because *C. philippinensis* is a less invasive tissue parasite than *S. stercoralis* [31] or because eosinophilic responses may vary with infection stage or chronicity. Therefore, eosinophilia is not a specific diagnostic marker for *C. philippinensis* infections. Some studies reported hypokalemia without concurrent hyponatremia [2, 22, 25, 27, 28, 31], suggesting variable degrees of electrolyte depletion depending on disease severity and duration.

Diagnostic approaches were dominated by stool microscopy, which was used in 10/17 case reports, 7/8 case series, and all observational studies (6/6), confirming its role as the primary diagnostic tool. However, the need for repeated stool examinations (2/17 case reports) highlights its limited sensitivity. Early infections may yield false-negative results due to intermittent egg shedding and low parasite loads in the stool [24]. The eggs of *C. philippinensis* have been reported to occur in two forms: thick-shelled eggs (peanut-shaped or swollen peanut-shaped) and thin-shelled eggs [28]. Earlier reports suggested that thin-shelled eggs are smaller than thick-shelled ones [41]. These thin-shelled eggs may contain larvae that hatch in the host intestine lumen, facilitating autoinfection and leading to hyperinfection [28]. In some cases, the eggs of *C. philippinensis* have been misidentified as those of *T. trichiura*, leading to an incorrect or delayed definitive diagnosis [5].

Histopathology and molecular testing served as important adjunctive diagnostic methods, particularly in stool-negative cases. Histopathological confirmation was reported in 1/17 case reports and 3/8 case series, while PCR was used in 2/17 case reports, including cases with negative stool microscopy. These findings highlight the added diagnostic value of alternative methods when routine stool examination is inconclusive. Histopathological abnormalities were predominantly described in case reports (8/17) and less consistently in case series (2/8), with villous atrophy and inflammatory infiltration emerging as the most common features (6/17 case reports). However, detection of parasite structures on histopathology was inconsistent, indicating limited sensitivity as a standalone diagnostic method. Histopathological findings showed that the parasite is most frequently found in the jejunum and occasionally the ileum, which may produce a characteristic ribbon-like appearance on imaging [14]. The histopathological examination of an intestinal biopsy should be the second-step diagnostic procedure if the stool examination is negative. However, the diagnostic procedure is difficult because the normal habitat of *C. philippinensis* is the jejunum, a part of the intestine where endoscopic examination is not feasible [15]. Aside from the small intestine, the parasite was reported in the colon, as demonstrated by endoscopic findings [19].

Co-infections were identified across all study designs, reported in 4/17 case reports, 4/8 case series, 1/2 prospective studies, and the case-control study, indicating that intestinal capillariasis commonly occurs in polyparasitic settings. Frequent co-infecting organisms included *G. lamblia*, *E. histolytica*, and *S. stercoralis*, which may complicate diagnosis and contribute to overlapping clinical features [20].

Treatment outcomes were consistently favorable. Clinical recovery was reported in 15/17 case reports and 5/8 case series, with both albendazole and mebendazole demonstrating comparable effectiveness. Albendazole achieved favorable outcomes in 8/17 case reports and 4/8 case series, while mebendazole showed similar success (6/17 case reports and 4/8 case series). These findings support the use of either agent as first-line therapy when administered for 10–30 days. Mortality was rare but reported (2/17 case reports and 1/8 case series), typically in severe or delayed cases.

Overall, the available evidence indicates that intestinal capillariasis remains a largely foodborne parasitic infection associated with the consumption of raw freshwater fish, while diagnostic challenges and limited community-based epidemiological data continue to obscure its true global burden. The synthesis of evidence across multiple study designs suggests that transmission is closely linked to cultural dietary practices involving raw freshwater fish. Strengthening food safety education, promoting behavioral change, and enhancing community-based awareness are critical to reducing disease burden. Improved diagnostic capacity, particularly repeated stool examinations combined with molecular or histopathological techniques, can facilitate earlier detection and better clinical outcomes.

Several limitations must be acknowledged. First, most of the available evidence derives from case reports and case series, which limit generalizability and increase susceptibility to publication bias. Second, geographic reporting was uneven, with a concentration of studies from selected regions in Asia and Africa and relatively few community-based prevalence studies. Consequently, the true population-level distribution of intestinal capillariasis remains uncertain. Third, molecular confirmation of infection was rarely reported, with most diagnoses relying on stool microscopy or histopathology. Variability in diagnostic methods and reporting practices across studies further limited direct comparison. These factors also introduce the risk of diagnostic misclassification, particularly in studies where confirmatory testing was incomplete. Future research should prioritize standardized diagnostic protocols, broader implementation of molecular confirmation, and well-designed community-based epidemiological studies to clarify the true prevalence, transmission dynamics, and public health impact of *C. philippinensis* infection.

## Conclusions

*Capillaria philippinensis* infection remains an underrecognized foodborne parasitic disease, predominantly reported from Asia and Africa. Transmission is strongly associated with the consumption of raw or undercooked freshwater fish. Clinical features were highly consistent across study designs, with chronic diarrhea, abdominal pain, weight loss, and hypoalbuminemia as hallmark findings. Diagnosis relies primarily on stool microscopy, although repeated examinations and adjunctive methods may be required. Treatment outcomes were generally favorable. Albendazole and mebendazole demonstrated comparable effectiveness across study designs. Mortality was rare but documented, emphasizing the need for early diagnosis. Overall, intestinal capillariasis is a foodborne infection with a consistent clinical profile. Improved food safety practices, awareness, and diagnostic capacity are essential to reduce disease burden and improve outcomes.

## Supplementary Information

Supplementary Material 1.

Supplementary Material 2.

Supplementary Material 3.

Supplementary Material 4.

## Data Availability

All data related to the present study are available in this manuscript, as well as in the Table S1, Table S2, and Table S3 files.

## Abbreviations

CT: Computed tomography
DNA: Deoxyribonucleic acid
EGD: Esophagogastroduodenoscopy
ICT: Immunochromatographic test
JBI: Joanna Briggs Institute
MeSH: Medical Subject Headings
PCR: Polymerase chain reaction
PECO: Population, Exposure, Comparator, and Outcomes
PRISMA: Preferred Reporting Items for Systematic Reviews and Meta-Analyses
PROSPERO: International Prospective Register of Systematic Reviews
SEM: Scanning electron microscopy
US: Ultrasonography

## Declarations

### Use of artificial intelligence

AI tools (ChatGPT) were used solely for language editing and clarity improvement. All scientific content, data analysis, and interpretations were performed by the authors, who take full responsibility for the manuscript.
