# Supplementary material for: Global distribution, clinical characteristics, and outcomes of human intestinal capillariasis, 2000–2025: a systematic review
Source: Infect Dis Poverty. 2026 Jun 15;15:68. doi: 10.1186/s40249-026-01464-3 (PMC13267653; doi:10.1186/s40249-026-01464-3)
Supplement: Supplementary file 1 — Supplementary Material 1. [file 40249_2026_1464_MOESM1_ESM.docx]

**Table S1. Search terms**

**General keywords**

“Capillaria philippinensis” OR Capillarias OR Skrjabinocapillaria OR Skrjabinocapillarias OR “Paracapillaria philippinensis” OR “Aonchotheca philippinensis” AND (human OR patients)

PubMed 19 August 2025

| No. | Key concept | Search terms | Results |
| --- | --- | --- | --- |
| 1. | *Capillaria philippinensis* | “Capillaria philippinensis”[MeSH Terms] OR Capillarias[MeSH Terms] OR Skrjabinocapillaria[MeSH Terms] OR Skrjabinocapillarias[MeSH Terms] OR “Paracapillaria philippinensis”[MeSH Terms] OR “Aonchotheca philippinensis”[MeSH Terms] OR “Capillaria philippinensis”[Text Word] OR Capillarias[Text Word] OR Skrjabinocapillaria[Text Word] OR Skrjabinocapillarias[Text Word] OR “Paracapillaria philippinensis”[Text Word] OR “Aonchotheca philippinensis”[Text Word] | 538 |
| 2. | Human | human[MeSH Terms] OR patients[MeSH Terms] OR human[Text Word] OR patients[Text Word] | 24,841,738 |
| 3. | 1 AND 2 | #1 AND #2 | 195 |

Embase 19 August 2025

| No. | Key concept | Search terms | Results |
| --- | --- | --- | --- |
| 1. | *Capillaria philippinensis* | 'capillaria philippinensis'/exp OR capillarias OR skrjabinocapillaria OR skrjabinocapillarias OR 'paracapillaria philippinensis'/exp OR 'aonchotheca philippinensis'/exp OR 'capillaria philippinensis':ti,ab,kw,de OR capillarias:ti,ab,kw,de OR skrjabinocapillaria:ti,ab,kw,de OR skrjabinocapillarias:ti,ab,kw,de OR 'paracapillaria philippinensis':ti,ab,kw,de OR 'aonchotheca philippinensis':ti,ab,kw,de | 112 |
| 2. | Human | 'human'/exp OR 'patients'/exp OR human:ti,ab,kw,de OR patients:ti,ab,kw,de | 31,967,410 |
| 3. | 1 AND 2 | #1 AND #2 | 89 |

Scopus 19 August 2025

| No. | Key concept | Search terms | Results |
| --- | --- | --- | --- |
| 1. | *Capillaria philippinensis* | TITLE-ABS-KEY ( "Capillaria philippinensis" OR Capillarias OR Skrjabinocapillaria OR Skrjabinocapillarias OR "Paracapillaria philippinensis" OR "Aonchotheca philippinensis" ) | 106 |
| 2. | Human | TITLE-ABS-KEY ( human OR patients ) | 30,594,088 |
| 3. | 1 AND 2 | 1 AND 2 | 91 |

Journal@Ovid 19 August 2025

| No. | Key concept | Search terms | Results |
| --- | --- | --- | --- |
| 1. | *Capillaria philippinensis* AND human | “Capillaria philippinensis” OR Capillarias OR Skrjabinocapillaria OR Skrjabinocapillarias OR “Paracapillaria philippinensis” OR “Aonchotheca philippinensis” AND (human OR patients) | 2 |

Nursing & Allied Health Premium 19 August 2025

| No. | Key concept | Search terms | Results |
| --- | --- | --- | --- |
| 1. | *Capillaria philippinensis* AND human | “Capillaria philippinensis” OR Capillarias OR Skrjabinocapillaria OR Skrjabinocapillarias OR “Paracapillaria philippinensis” OR “Aonchotheca philippinensis” AND (human OR patients) | 19 |

Web of Science 21 August 2025

| No. | Key concept | Search terms | Results |
| --- | --- | --- | --- |
| 1. | *Capillaria philippinensis* AND human | “Capillaria philippinensis” OR Capillarias OR Skrjabinocapillaria OR Skrjabinocapillarias OR “Paracapillaria philippinensis” OR “Aonchotheca philippinensis” AND (human OR patients) | 55 |

Google Scholar 19 August 2025

| No. | Key concept | Search terms | Results |
| --- | --- | --- | --- |
| 1. | *Capillaria philippinensis* AND human | Capillaria philippinensis | Screening only the first 200 articles (all records, 1,800) |
