# Supplementary material for: Global distribution, clinical characteristics, and outcomes of human intestinal capillariasis, 2000–2025: a systematic review
Source: Infect Dis Poverty. 2026 Jun 15;15:68. doi: 10.1186/s40249-026-01464-3 (PMC13267653; doi:10.1186/s40249-026-01464-3)
Supplement: Supplementary file 4 — Supplementary Material 4. [file 40249_2026_1464_MOESM4_ESM.docx]

**Supplementary Text S1. Life cycle of *Capillaria philippinensis***

The life cycle of *Capillaria philippinensis* involves freshwater fish as intermediate hosts and humans as definitive hosts. Adult worms inhabit the small intestine of infected humans, where female worms produce both thick-shelled and thin-shelled eggs. Thick-shelled eggs are excreted in feces into freshwater environments. Under suitable environmental conditions, these eggs embryonate and hatch, releasing larvae that are ingested by small freshwater fish. Within the fish host, larvae develop into infective third-stage larvae (L3) ^1^.

Humans acquire infection through ingestion of raw or undercooked freshwater fish containing infective larvae. After ingestion, larvae are released in the small intestine, penetrate the intestinal mucosa, and mature into adult worms. Female worms subsequently produce eggs within the intestinal tract ^1^. Thin-shelled eggs may embryonate and hatch within the human intestine, releasing larvae that reinvade the intestinal mucosa, resulting in autoinfection and potentially severe hyperinfection if untreated ^1^. Piscivorous birds have been proposed as potential reservoir hosts because they may consume infected fish and excrete parasite eggs into freshwater environments; however, natural infection in birds has not been definitively confirmed ^1^.

**References**

1. CDC. Capillariasis (Intestinal and Hepatic) — *Capillaria*, *Capillaria philippinensis*/*C. hepatica.* 2024. Available from: <https://www.cdc.gov/capillaria/about/index.html>. Accessed 25 November 2025.
